# Phytochemical Profiling and Quality Control of *Terminalia sericea* Burch. ex DC. Using HPTLC Metabolomics

**DOI:** 10.3390/molecules26020432

**Published:** 2021-01-15

**Authors:** Nduvho Mulaudzi, Chinedu P. Anokwuru, Sidonie Y. Tankeu, Sandra Combrinck, Weiyang Chen, Ilze Vermaak, Alvaro M Viljoen

**Affiliations:** 1 Department of Pharmaceutical Sciences, Faculty of Science, Tshwane University of Technology, Private Bag X680, Pretoria 0001, South Africa; MulaudziN@tut.ac.za (N.M.); anokwurucp@tut.ac.za (C.P.A.); tankeus@tut.ac.za (S.Y.T.); combrincks@tut.ac.za (S.C.); chenw@tut.ac.za (W.C.); vermaaki@tut.ac.za (I.V.); 2 SAMRC Herbal Drugs Research Unit, Faculty of Science, Tshwane University of Technology, Private Bag X680, Pretoria 0001, South Africa

**Keywords:** *Terminalia sericea*, quality control, metabolomics, rTLC, HPTLC

## Abstract

*Terminalia sericea* is used throughout Africa for the treatment of a variety of conditions and has been identified as a potential commercial plant. The study was aimed at establishing a high-performance thin layer chromatography (HPTLC) chemical fingerprint for *T. sericea* root bark as a reference for quality control and exploring chemical variation within the species using HPTLC metabo3lomics. Forty-two root bark samples were collected from ten populations in South Africa and extracted with dichloromethane: methanol (1:1). An HPTLC method was optimized to resolve the major compounds from other sample components. Dichloromethane: ethyl acetate: methanol: formic acid (90:10:30:1) was used as the developing solvent and the plates were visualized using 10% sulfuric acid in methanol as derivatizing agent. The concentrations of three major bioactive compounds, sericic acid, sericoside and resveratrol-3-*O*-*β*-rutinoside, in the extracts were determined using a validated ultra-performance liquid chromatography-photodiode array (UPLC-PDA) detection method. The rTLC software (written in the R-programming language) was used to select the most informative retardation factor (R*f*) ranges from the images of the analysed sample extracts. Further chemometric models, including principal component analysis (PCA) and hierarchical cluster analysis (HCA), were constructed using the web-based high throughput metabolomic software. The rTLC chemometric models were compared with the models previously obtained from ultra-performance liquid chromatography coupled with mass spectrometry (UPLC-MS). A characteristic fingerprint containing clear bands for the three bioactive compounds was established. All three bioactive compounds were present in all the samples, although their corresponding band intensities varied. The intensities correlated with the UPLC-PDA results, in that samples containing a high concentration of a particular compound, displayed a more intense band. Chemometric analysis using HCA revealed two chemotypes, and the subsequent construction of a loadings plot indicated that sericic acid and sericoside were responsible for the chemotypic variation; with sericoside concentrated in Chemotype 1, while sericic acid was more abundant in Chemotype 2. A characteristic chemical fingerprint with clearly distinguishable features was established for *T. sericea* root bark that can be used for species authentication, and to select samples with high concentrations of a particular marker compound(s). Different chemotypes, potentially differing in their therapeutic potency towards a particular target, could be distinguished. The models revealed the three analytes as biomarkers, corresponding to results reported for UPLC-MS profiling and thereby indicating that HPTLC is a suitable technique for the quality control of *T. sericea* root bark.

## 1. Introduction

It has been estimated that southern Africa is home to 30,000 species of Higher Plants, of which roughly 24,000 are indigenous to South Africa [1]. Approximately 3000 of these species are used as medicines, and records indicate that at least 350 species are commonly used and traded as medicinal plants [1]. Although South Africa has developed a large diversity of herbal products, only a few indigenous plants have been standardized for commercial purposes [2]. Quality control (QC) therefore remains a major stumbling block to commercialization of herbal drugs, due to the lack of standardized protocols for the analysis of raw materials and products. Marker compounds that can be used for species verification and to evaluate the chemical consistency of raw materials and products, have not been identified for the majority of plant species with commercial potential [3].

Currently, a variety of chromatographic fingerprinting techniques are available for the QC of herbal products. These include highly sophisticated techniques such as gas chromatography (GC) with mass spectrometry (MS) and/or other detection systems, and high-performance- (HPLC) or ultra-performance liquid chromatography (UPLC) coupled with MS or other detection systems [4]. However, planar chromatography techniques, specifically thin layer chromatography (TLC) and high-performance TLC (HPTLC), are becoming increasingly popular for QC due to their speed and suitability to commercial environments [5,6]. High-performance thin layer chromatography (HPTLC) is a readily available semi-automated technique used for rapid-throughput screening of samples to authenticate herbal products and to differentiate between related herbal species [7]. One of the major advantages of HPTLC resides in its flexibility to optimize operational parameters that include sample application and plate development, documentation and derivatization [8]. Compared to manual TLC, the technique yields better reproducibility, resolution and sensitivity, particularly when combined with improved digital scanning and documentation software, resulting in a more complete extraction of the information. In combination with chemometric data analysis, HPTLC has been reported to be a powerful tool for statistical analysis of the chemical profiles of herbal medicines, and for exploring differences and similarities within individual samples [9,10,11]. The development of rTLC software, available as an open-source web package for image processing, presents new opportunities for chemometric analysis of HPTLC chromatograms. It is the first fast and simple modelling technique that is compatible with planar chromatography, offering useful features for the analysis of HPTLC data, such as band comparison, signal pre-processing, as well as the comma separated value (CSV) application [12]. Application of the software converts HPTLC data to a 3D array, in which rows represent samples, columns represent the retardation factor (R*f*) and layers are related to different channels (red/green/blue (RGB) and greyscale). These layers can be separated and converted to four sets of two-dimensional data. This allows pattern recognition techniques, including principal component analysis (PCA), hierarchical cluster analysis (HCA), and heat maps to be applied to each individual channel. Prediction techniques such as random forest (RF), linear discriminant analysis (LDA), support vector machine (SVM), partial least-squares (PLS), and classification and regression tree (CART) analysis, are integrated into the software. 

The root-bark of *Terminalia sericea* Burch. ex DC. (family Combretaceae) is a popular traditional remedy used throughout Africa for the treatment of conditions such as diarrhea, cough, venereal diseases and tuberculosis [13]. It has been identified as a species with excellent potential for commercialization [14]. Phytochemical studies have revealed the presence of hydrolysable tannins (ellagic acid, flavogallonic acid dilactone, methyl-flavogallonate) lignans (anolignan b, termilignan b), stilbenes (resveratrol-3-*O*-β-rutinoside, resveratrol-3-(6′’-galloyl)-*O*-β-*d*-glucopyranoside), flavonoids (quercetin-3-(2′′- galloylrhamnoside) and triterpenoids (arjunic acid, sericic acid, arjunglucoside I, sericoside, arjunetin) in the roots of *T. sericea* [15,16,17,18,19,20]. In a recent study [20], the chemical variability within *T. sericea* root bark samples representing several populations in Limpopo Province, South Africa, was attributed mainly to sericic acid, sericoside and resveratrol-3-*O*-*β*-rutinoside. The current study was aimed at establishing a characteristic HPTLC chemical fingerprint for *T. sericea* root bark for quality control purposes. In addition, the availability of rTLC provided a powerful tool for exploring the chemical variation of the major compounds within wild populations.

## 2. Results and Discussion

### 2.1. Chemical Fingerprinting to Determine Inter- and Intra-Population Chemical Variation within Root Bark

**Figure 1:**
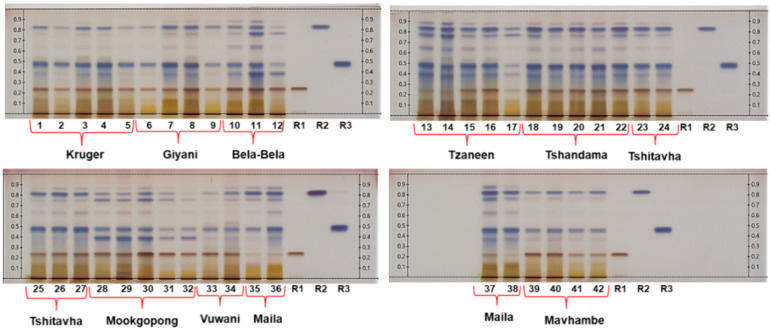
HPTLC fingerprints of the dichloromethane: methanol (1:1) extracts of *T. sericea* samples from 10 different localities (Tracks 1–42). The extracts were applied at the same concentration and the plates were developed using dichloromethane: ethyl acetate: methanol: formic acid (90:10:30:1). Three standards (Track R1 (resveratrol-3-*O*-*β*-rutinoside), Track R2 (sericic acid) and Track R3 (sericoside) were included on the plates, which were viewed under white light after derivatisation with 10% sulfuric acid in methanol.

Dichloromethane: ethyl acetate: methanol: formic acid (90:10:30:1) and 10% sulfuric acid in methanol were identified as the most suitable mobile phase and derivatizing agent, respectively, for HPTLC fingerprinting of *T. sericea*, after repeatedly analysing randomly selected extracts using an assortment of developing solvents and applying a variety of derivatizing agents. The use of white light was found to be the most suitable for visualizing the compounds, as compared to ultraviolet radiation at 254 and 366 nm. The chemical variation within 42 root bark samples, collected from 10 different localities, was determined by carefully examining their HPTLC profiles. Images of the developed and derivatized plates obtained under white light are presented in Figure 1. Differences in the intensities of the bands representing the major compounds are evident within samples from different localities (for example, Kruger and Maila samples), as well as within samples from the same locality, for example Mookgophong samples P3 and P4 (Tracks 30 and 31). Inspection of the HPTLC plates revealed that resveratrol-3-*O*-*β*-rutinoside (R1), sericic acid (R2) and sericoside (R3) are major constituents of the root bark extracts, confirming previous reports [15,20]. Differences in their concentrations are evident from the different band intensities. Qualitative analysis of the major compounds indicated that samples from two localities, namely Tzaneen (Z1, Z2 and Z5) (Tracks 13, 14 and 17) and Maila (J1, J2 and J4) (Tracks 35, 36 and 38) contain the lowest concentrations of resveratrol-3-*O*-*β*-rutinoside, since the band intensities (brown band) are very low at the corresponding R*f* value of 0.24. Faint bands representing low concentrations of sericic acid, a blue band at R*f* 0.83, are visible in samples from Bela-Bela (B3) (Track 12), Vuwani (V1) (Track 33) and Mookgophong (P4 and P5) (Tracks 31–32). Sericoside, at R*f* 0.48, can be observed in low concentrations in samples from Tzaneen (TZ) (Track 17), Bela-Bela (B3) (Track 12) and Mookgophong (P4 and P5) (Tracks 31–32). 

Despite some differences in band intensities, all of the fingerprints reflected the same basic features. It can therefore be concluded that a characteristic *T. sericea* root bark fingerprint for species authentication should contain clear bands representing resveratrol-3-*O*-*β*-rutinoside, sericic acid and sericoside. The commercial potential of *Terminalia sericea* as an anti-infective agent has not been fully exploited and this has been attributed to a lack of standardisation and quality control [20]. The reported Rf values and colours of the bands can be used for easy identification of the compounds in extracts of *T. sericea* root bark. Recently, Sotenjwa and co-workers [21] reported such information to facilitate the identification of compounds including acacetin, scopoletin and scopolin in *Artemisia afra* for quality control purposes. In another study [22], three hydroxy methoxy flavones and a coumarate were identified as markers for quality control of *Athrixia phylicoides* (Bush tea). 

### 2.2. Quantitative Analysis

A UPLC-photodiode array detection (PDA) method was validated according to ICH guidelines [23], and used for the quantitative determination of the three standards. The results of the method validation are presented in the Supplementary Material (Table S1). The calibration curves for the three compounds were all characterized by a high regression coefficient (≥0.998), indicating a good relationship between detector response (peak areas) and the tested concentration range (0.500–100 µg/mL) at a 95% confidence level. The limits of detection (LODs) for sericic acid and resveratrol-3-*O*-β-rutinoside were comparable (25.2 and 23.3 ng/mL) and higher than that obtained for sericoside (11.6 ng/mL). The accuracy of the extraction method was satisfactory since high recoveries (98–102%) were determined for the three compounds. Recoveries of between 80 and 120% are regarded as acceptable [24]. The small percentage relative standard deviations (RSDs) obtained for intra- and interday precision reflect good instrument repeatability and indicate stability of the three compounds in the samples. 

The intensities of the bands representing each of the three compounds were approximated through visual inspection of the HPTLC chromatograms (Figure 1) as faint (+), intense (++) or very intense (+++). This was done independently of the UPLC-PDA data to prevent bias. The results are shown in Table 1. Samples from Tzaneen (TZ1 3.03 mg/g), TZ2 (2.22 mg/g) and TZ5 (2.23 mg/g) and Maila (J1 (1.54 mg/g), J2 (1.43 mg/g) and J4 (2.65 mg/g), all classified as (+), contained the lowest concentrations of resveratrol-3-*O*-*β*-rutinoside within their respective localities, as well as the least compared to all localities. The highest concentration (29.8 mg/g) was recorded for a sample from Mookgophong (Track 30), assigned (+++) after visual inspection of the plates. Sericic acid was present in low concentrations in Tzaneen (TZ5 (4.81 mg/g; +), Bela-Bela (BP3 (2.58 mg/g; +), Mookgophong (MP4 (5.25 mg/g; +) and MP5 (5.02 mg/g; +) samples, with one Tshandama (Track 18) sample containing the highest concentration of sericic acid (22.7.mg/g; +++) overall. Sericoside was reported in low concentrations in samples from KNP (K2 (6.14 mg/g; +), Bela-Bela (BP3 (4.82 mg/g; +), Mookgophong (MP4 (5.23 mg/g; +) and (MP5 (7.55 mg/g; +) and Vuwani (V1 (6.89 mg/g; +), with the highest concentration reported to be 30.16 mg/g from Tzaneen (Track 13; +++). Samples from Tshandama (TSA1-TSA5) (Tracks 18–22) and Tshitavha (TSH1–TSH5) (Tracks 23–27) contained high concentrations of all the major compounds, as reflected by the intense bands corresponding to the standards in these samples (all allocated +++). The congruent results between the quantitative analysis and the band intensities prove that the HPTLC method is reliable for QC (qualitative and quantitative) of *T. sericea*. It is feasible that semi-quantitative analysis, involving the application of increasing concentrations of the standards to a plate and comparing their band intensities to those in the samples, would provide a sufficiently accurate estimate of the compound concentrations for QC purposes.

### 2.3. Chemometric Analysis of HPTLC Data

#### 2.3.1. Selection of Appropriate R*f* Ranges for Modelling

**Figure 2:**
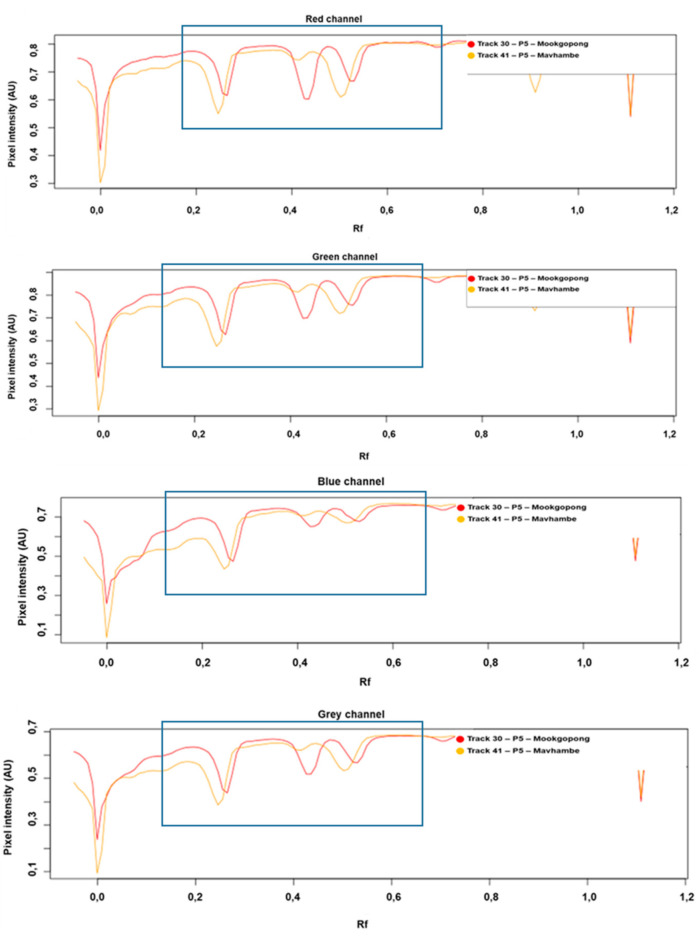
HPTLC tracks and the corresponding rTLC densitograms of *T. sericea* samples from Mookgophong (Track 30) and Mavhambe (Track 41), depicting different channels and R*f* ranges that reveal differences between the samples. The blue rectangles indicate the R*f* range selected for each sample for each channel. These channels and ranges were used as variables to construct the PCA model.

Plots of pixel intensity as a function of the R*f* (retardation factor) value (densitograms), obtained from individual sample fingerprints using the rTLC software, are presented in Figure 2. Two samples, displaying very different band intensities, Mookgophong (Track 30) and Mavhambe (Track 41) (Figure 2), were selected to identify the channel that best reflects differences between the two samples, so that they can be clearly differentiated. A channel in this case represents a digital image represented by just one primary colour (Red, Green, Blue) or the grayscale (average) image. It was observed that for all of the channels, specific ranges contributed to the observed variations between the samples. All four channels (red, blue, green and grey) indicated differences in the compound intensities over the R*f* range 0.2 to 0.7, as indicated by the blue rectangles (Figure 2).

#### 2.3.2. Chemical Variation within T. *sericea* Root Bark Samples from Three Districts in Limpopo Province, South Africa

Visual inspection of the HPTLC plates revealed different band intensities for the three major constituents (resveratrol-3-*O*-*β*-rutinoside, sericic acid, and sericoside) present in *T. sericea* root bark extracts. This visual observation motivated the use of chemometric models to establish if samples from the Mopani (Giyani, Tzaneen), Waterberg (Bela-Bela, Mookgophong) and Vhembe (Maila, Vuwani, Mavhambe, Kruger, Tshandama, Tshitavha) districts representing distinctly different geographical localities in the Limpopo Province, could be chemically distinguished. The chemometric models obtained from the rTLC were compared with previous models obtained from UPLC-MS data [20]. 

**Figure 3:**
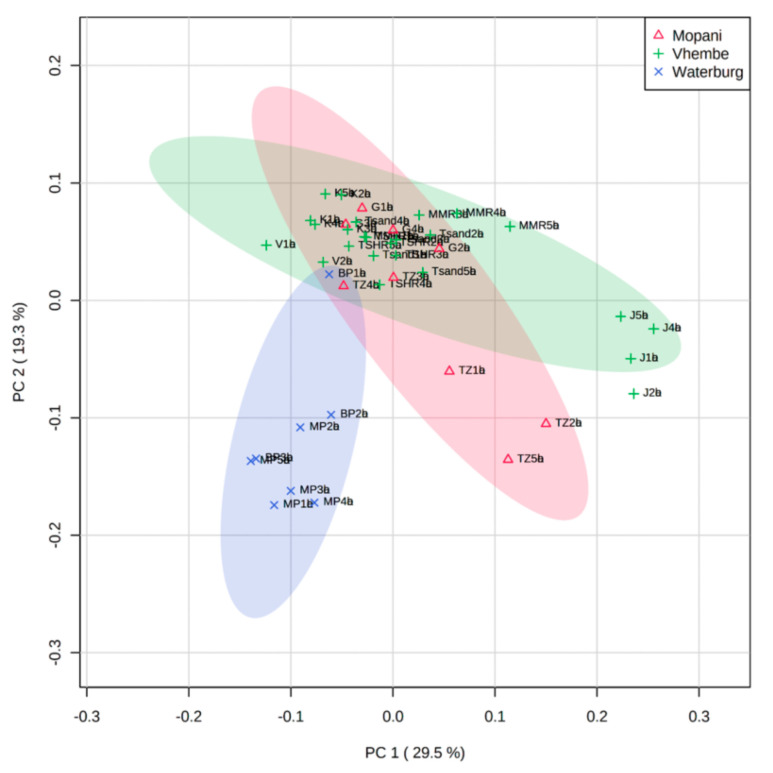
PCA scores plot derived from HPTLC data and coloured according to the three districts where the samples were collected.

An eight-principal component PCA model was constructed using the fingerprints of the root bark samples from the three districts, to explore trends within the analytical dataset. A scores plot was constructed using the first and second components, accounting for 48.9% of the variation in the data. The scores plot was coloured according to the three districts (Figure 3). Generally, three main clusters can be observed that are related to the three districts. However, closer investigation of the three clusters indicate some extent of variation between and within districts. The Mopani (Red) cluster, encompassing samples collected from two localities (Giyani (G) and Tzaneen (TZ)), indicate that the chemistry of three TZ samples (TZ1, 2, 5) differs from that of other samples. Similarly, samples from Maila (J1–J4) are different from other Vhembe district samples (green). All samples from Waterberg district (blue), except BP1, cluster together and are separated from samples from the other districts. It can therefore be concluded that samples are not strictly clustered according to the district of origin, indicating differences within the chemistry of samples from the same district.

**Figure 4:**
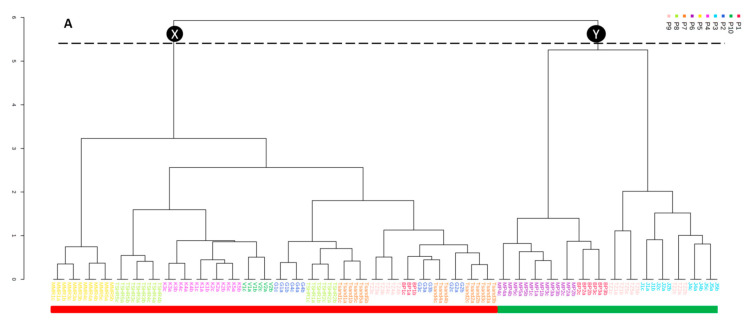

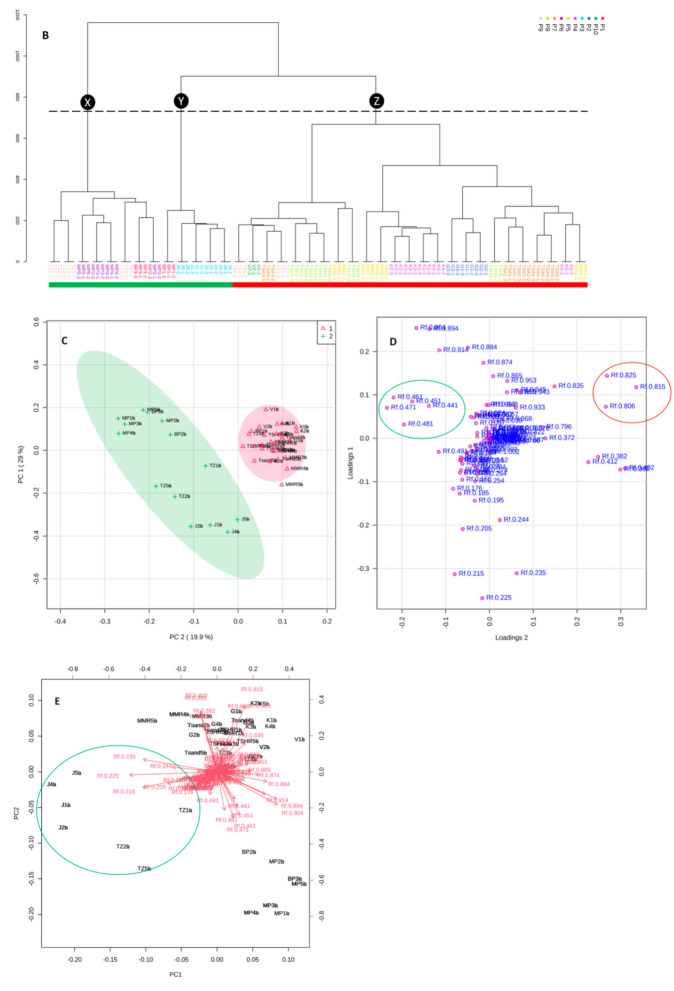
(**A**) Dendrogram obtained from the HCA of the rTLC data, emphasizing the chemical consistency within the populations, (**B**) dendrogram obtained from the HCA of the UPLC-MS data, with a clustering pattern similar to that obtained with the HPTLC data (**A**) although samples J1–J4 and BP2 and 3 (Branch Y) are clustered separately from MP1-MP5 and TZ1, 2 and 5 (Branch X), (**C**) the scores plot coloured according to the HCA (HPTLC data), (**D**) the loadings plot indicating potential markers (HPTLC data), and (**E**) the biplot (HPTLC data) indicating the association of compounds with samples (resveratrol-3-*O*-*β*-rutinoside to samples J1–J4 and TZ1–2 and TZ5). Samples found in the green clusters (**A**–**E**) have a high concentration of sericic acid (except samples J1–J4) while samples within the red cluster contain a high concentration of sericoside.

The inter- and intra-district variation in the chemistry that was observed encouraged further investigation of the ten populations (Tzaneen, Giyani, Maila, Tshitavha, Vuwani, Mavhambe, Tshandama, Kruger, Bela-Bela and Mookgophong) in an individual manner, to explore potential chemotypes based on localities. A dendrogram (Figure 4A) was constructed from the HCA to assess inter- and intrapopulation variation in the chemistry and to identify potential chemotypes based on locality. Two major branches, X and Y, can be observed on the dendrogram that are related to the separation along PC2 (Figure 3), with the X branch recording samples on the positive PC2, while the Y branch regroups samples on the negative PC2 (Figure 3). Branch X consists of samples from Kruger (K), Giyani (G), Vuwani (V), Tzaneen (TZ 3 and 4), Tshandama (TSA), Mavhambe (MMR), Bela-Bela (BP1) and Tshitavha (TSH), while Branch Y comprises samples from Mookgophong (MP), Bela-Bela (BP2 and 3) and Maila (J1–J4). Samples from Mavhambe (MMR), Kruger (K), Vuwani (V), Mookgophong (MP) and Bela-Bela (BP2 and 3) all cluster according to population. Notably, the clustering pattern is similar to the one obtained with the UPLC-MS data (Figure 4B). However, in the UPLC-MS data, samples J1–J4 and BP2 and 3 (Branch Y) were clustered separately from MP1-MP5 and TZ1, 2 and 5 (Branch X). From the HPTLC data, the separation of TZ3 and 4 from TZ1, 2 and 5 could be related to the differences in the band intensities (concentrations) of resveratrol-3-*O*-*β*-rutinoside, as previously indicated by the qualitative and quantitative data. This plausible explanation for the separation of the samples could not be deduced from the UPLC-MS data alone (Figure S1A,B), suggesting that HPTLC analysis is able to provide information that may be hidden when using liquid chromatography-mass spectrometry (LC-MS) for QC studies. The two branches defined by the dendrogram were used for class assignment: samples on Branch X were assigned to Chemotype 1 and samples on Branch Y to Chemotype 2. The scores plot was later coloured according to the two chemotypes (Figure 4C). The constructed loadings plot (Figure 4D) indicates some of the R*f*s that contribute to the clustering observed on the dendrogram. Taking into account that the rTLC software calculates the horizontal mean for each pixel of the chromatogram, the major compounds of *T. sericea* (resveratrol-3-*O*-*β*-rutinoside, sericic acid and sericoside) were indicated within a range of R*f* values. Therefore, the brownish resveratrol-3-*O*-*β*-rutinoside band is defined by the R*f* range 0.166–0.235; the blue-ish band representing sericic acid is defined by the R*f* range 0.815–0.865, and the blue band sericoside appears within the R*f* range 0.431–0.52. Considering the ranges indicated for each of the three major compounds, the loadings plot revealed that sericic acid and sericoside contributed to the clustering (Figure 4A). This trend was similar to the observation of the previously published UPLC-MS data [20] (Figure S1B).

In addition, pertinent R*f* values (0.244, 0.205, 0.215, 0.235 and 0.225) (Figure 4D) corresponding to resveratrol-3-*O*-*β*-rutinoside on the HPTLC plates, were observed along the negative loadings 1 (Figure 4D). This observation could be related to additional variation along PC1, as illustrated in Figure 4C. To further establish the association of these prominent values to the corresponding samples, a bi-plot was constructed (Figure 4E). The bi-plot (Figure 4E) indicates how samples are correlated with R*f* values. The plot revealed that the R*f* values allocated to resveratrol-3-*O*-*β*-rutinoside were related to the J samples and a few TZ samples (TZ1, TZ2 and TZ5). The association of resveratrol-3-*O*-*β*-rutinoside with J and TZ samples correlates with the qualitative and quantitative observations.

Taking into account that the major compounds targeted in the study were not visible under 366 nm, the plate were viewed under white light. According to Viennot and Hosson [25], the background of an HPTLC track without visible bands displays the highest intensity under white light when compared to regions where bands are present. Therefore, in the densitogram, the amplitude of the white background of each track used in this experiment should be higher compared to the amplitudes corresponding to coloured bands (compounds) revealed on the plate. In our study, the amplitudes of the bands on the densitogram corresponding to the bands displayed on the plate were lower than that of the background (where there was no visible band). When the densitograms of the three standards (resveratrol-3-*O*-*β*-rutinoside, sericic acid, sericoside) samples were constructed, the amplitudes of the bands for the three standards were found to be lower than that of the white background (which contained no sample), resulting in a negative densitogram. This confirmed that there is a negative correlation between the loadings plot and the band intensities. From the loadings plot (Figure 4D) it appears that sericic acid indicated by the red circle (R*f* 0.825, 0.815, 0.806) is concentrated in Chemotype 1, while sericoside indicated by the green circle (R*f* 0.461, 0.471, 0.451, 0.441, 0.481) is concentrated in Chemotype 2. However, this observation turned out to be a negative correlation in the data obtained, as explained above and when compared to the UPLC-MS data. In actual fact, sericoside was concentrated in Chemotype 1, while sericic acid was concentrated in Chemotype 2. In addition, the loadings plot (Figure 4D) indicated that resveratrol-3-*O*-*β*-rutinoside (R*f* 0.244, 0.205, 0.215, 0.235, 0.225) is present in high concentrations in the J samples and TZ1, TZ2 and TZ5. However, the qualitative and quantitative data indicate that the concentration of resveratrol-3-*O*-*β*-rutinoside is low in these samples. Considering the negative correlation principle, resveratrol-3-*O*-*β*-rutinoside is actually low in J samples and TZ1, TZ2 and TZ5 and now in agreement with the quantitative and qualitative data. This observation is consistent with the findings when using UPLC-MS data (Figure S1). In other words, sericoside is associated with Chemotype 1 (samples from Kruger, Giyani, Vuwani, Tzaneen (TZ 3, 4), Tshandama, Mavhambe, Bela-Bela (BP1) and Tshitavha) while sericic acid is associated with Chemotype 2 (samples TZ1, 2 and 5 from Tzaneen, Maila, Mookgophong and Bela-Bela-BP2 and 3). Both the UPLC-MS and HPTLC techniques indicated the existence of two chemotypes, defined by sericoside and sericic acid, respectively, within *T. sericea* samples from Limpopo Province. Both techniques were also used for the QC of *Xysmalobium undulatum* [26]. Other studies have reported the potential for the application of rTLC for QC. “Imphepho” species (*Helichrysum odoratissimum* and *Helichrysum petiolare*), which are commonly used interchangeably, were differentiated using rTLC, and interpopulation differences were identified within the individual species [27]. In another study [21], chemotypes of the non-volatile constituents of *Artemisia afra* (African wormwood), possibly with different therapeutic properties, were identified using rTLC. The use of the rTLC package for chemometric studies is relatively recent and the applications described in literature are therefore limited. One of the goals of QC is to associate chemotypes with biological activity. The identification of biomarkers is an important component of the process of herbal formulation. Besides their use to verify the authenticity of plant material, the presence of compounds associated with the biological activities are an indicator of the efficacy of a product. In *T. sericea* root bark, sericoside and sericic acid were previously identified as biomarkers [28,29]. Sericoside is known to possess anti-inflammatory and antiaging activities [30,31,32] while sericic acid is known as an antibacterial agent [20]. Therefore, sampling from Vhembe district would likely be a preferred choice to Waterberg district for samples to be used in products related to these activities. However, for raw materials destined for products with antibacterial activities, sampling from Mopani and Waterberg districts would be preferred compared to Vhembe district (except samples from Maila), since they contain higher concentrations of sericic acid or lower concentrations of resveratrol-3-*O*-*β*-rutinoside. It is evident that HPTLC can be used for quality control of *T. sericea* root samples. 

## 3. Materials and Methods

### 3.1. Plant Material and Extraction

*Terminalia sericea* root samples (*n* = 42), collected from ten different locations in the Limpopo Province of South Africa (Table 1), were washed, air-dried for a week and then powdered using a Sunbeam^®^ coffee grinder. The trees were identified by Prof Peter Tshisikhawe (Department of Botany, University of Venda). For each population, a voucher specimen was prepared and deposited in the herbarium of the Department of Botany. Samples were also sourced from a vendor in Thohoyandou mentioning that the material was obtained in the vicinity of the Kruger National Park (hence referred to as Kruger samples). A 1.0 g portion of each powder was extracted with 10.0 mL of dichloromethane: methanol (1:1) for 10 min using an ultrasonic bath (ULTRA-SONIC, LIBMB, Labcon, Krugersdorp, South Africa), where after the extract was filtered through a 0.45 μm nylon syringe filter (Acrosdisc^®^, Pall, New York, NY, USA) prior and transferred to individual autosampler vials for HPTLC analysis.

### 3.2. Chemicals and Reference Standards

Organic solvents (dichloromethane, ethyl acetate and methanol), used for extraction and mobile phase optimization, were of analytical grade (Thembane Chemicals, Johannesburg, South Africa). Concentrated formic acid (85.0% *w*/*w*) and sulfuric acid (95.0% *w*/*w*) were purchased from the same supplier. Reference standards used for the quantitative determination and for quality control purposes, namely resveratrol-3-*O*-*β*-rutinoside (purity = 94.1%), sericic acid (purity = 99.0%), and sericoside (purity = 99.0%), were previously isolated [20]. 

### 3.3. Analysis of Root Bark Samples Using HPTLC

A 2 µL volume of each of the dichloromethane: methanol (1:1) extracts (100 mg/mL dissolved in methanol), along with the reference standards (1 mg/mL in methanol) were applied to Silica gel 60 F254 (20 × 10 cm^2^) glass plates (Merck Ltd., Germany). Dichloromethane: ethyl acetate: methanol: formic acid (90:10:30:1) was optimized and selected as the preferred developing solvent. The chamber was saturated for 20 min at 33% relative humidity and 23 ± 2 °C using 25 mL of the solvent. For development, the solvent (10 mL) was allowed to migrate to a distance of 70 mm. Visualization of the compounds was done by spray-application of 10% sulfuric acid in methanol, which was followed by heating the plate for 3 min at 100 °C. It was then left at room temperature for 3 min to cool. The major compounds targeted in this study were not visible under 256 nm and 366 nm but viewing under white light reflectance revealed their presence. 

### 3.4. Method Validation

A UPLC-photodiode array (PDA) method was developed for the simultaneous quantification of resveratrol-3-*O*-*β*-rutinoside, sericic acid and sericoside in all 42 samples. The method was validated for linearity, accuracy and precision. The limits of detection (LOD) and quantification (LOQ) for each standard were calculated, following the construction of a calibration curve (0.500–100 μg/mL) and regression analysis of the data [23]. The accuracy of the method was determined by evaluating the recovery of the three standards from an extract solution. The extract, containing a known concentration of each standard, was spiked with 2.50, 25.0 and 100 μg/mL of the standard solution in triplicate. The instrument precision was determined by intra- and inter-day analysis to establish the reproducibility of the method. A solution of each standard (10 μg/mL) was analysed three times daily, at different time intervals, over three days. The inter-day precision analysis was done by determining the relative standard deviation (RSD) of the means obtained on each day.

### 3.5. Analysis of Root Bark Samples Using UPLC-PDA and UPLC-MS

The UPLC-MS data was used for chemometric analysis as described in [11], while UPLC-PDA was used to determine the concentrations of the three standards, following validation of the method using ICH guidelines [23]. The root extracts were analysed using a Waters Acquity Ultra Performance Liquid Chromatography system equipped with a photodiode array (PDA) detector (Waters, Milford, MA, USA) and interfaced with a Xevo G2QToF MS (Waters, Milford, MA, USA). The injection volume was 2 μL (full loop injection). Chromatographic separation was achieved on an Acquity UPLC BEH C18 column (150 mm × 2.1 mm, i.d., 1.7 μm particle size, Waters) maintained at a constant temperature of 30 °C. The mobile phase consisted of 0.1% aqueous formic acid (Solvent A) and UPLC grade (Microsep, USA) acetonitrile (Solvent B) at a flow rate of 0.3 mL/min. Gradient elution was executed as follows: the initial ratio was 90% A:10% B, changed to 50% A:50% B within 4 min, to 50% A:50% B in 6 min, to 5% A:95% B in 2.5 min, maintaining for 0.5 min, before returning to the initial ratio in 0.5 min. The system was equilibrated for 2 min between consecutive runs. A Micromass–LCT Premier quadrupole-Time-of-Flight-mass spectrometer (QToF-MS) (Waters, Milford, MA, USA) was hyphenated with the UPLC, using the same conditions as before. Both positive and negative electrospray ionization (ESI) modes were evaluated, but the positive mode resulted in a greater abundance of ions and provided spectra with more information. Therefore, the MS was further operated in the positive mode. Nitrogen (600 L/h) was used as the desolvation gas and the desolvation temperature was maintained at 400 °C. Data were acquired between *m/z* 100 and 1200. The following settings were used for the mass spectrometer: capillary voltage 3500 V; sampling cone voltage 38 V; source temperature 100 °C. 

### 3.6. Chemometric Analysis of the HPTLC Data Using rTLC 

After HPTLC analysis, rTLC software (Version 4.0, Xia Lab, McGill University, Montreal, Quebec, Canada) (written in the R-programming language) was used to extract data points (Excel file) from HPTLC plates [12]. A set of chromatographic images, obtained from the HPTLC analysis of the 42 root bark samples were simultaneously uploaded to the software and converted to a numerical data matrix for analysis. The software supports the uploading of images in JPEG, TIFF and PNG format [12]. The RGB colour channels, as well as the greyscale channel, which represents the average of the three other channels, were investigated to select the channels that will best represent the variation within *T. sericea*. In addition, plots (densitograms) of peak intensities were constructed as a function of the migration distance (R*f*). These densitograms assisted with the selection of R*f* ranges that reflected bands associated with differences and similarities within the samples. After selection of the most informative R*f* ranges, the corresponding data points (Excel file comprising a data matrix of 126 samples × 102 compounds) was exported for further analysis using MetaboAnalyst 4.0 (Xia Lab, McGill University, Montreal, QC, Canada) (www.metaboanalyst.ca/MetaboAnalyst/home.xhtml) [33], an online high throughput metabolomic software. Quantile normalization was applied to the data and different methods were explored by applying unsupervised PCA and HCA. This was done to establish chemical variation within *T. sericea* root bark collected from ten locations representing three districts in Limpopo Province, South Africa.

## 4. Conclusions

A characteristic HPTLC fingerprint, featuring resveratrol-3-*O*-*β*-rutinoside, sericic acid and sericoside, was established for *T. sericea* root bark using dichloromethane: ethyl acetate: methanol: formic acid (90:10:30:1) as the developing solvent and 10% sulfuric acid as visualization reagent. Inter- and intrapopulation quantitative, rather than qualitative variation, was evident in the root bark samples collected from the 10 localities. The combination of HPTLC and chemometrics, employed to further investigate the chemical variation within the root bark samples, revealed that clustering was mostly population specific, since samples from the same population clustered closely. The HPTLC analysis confirmed the quantitative differences between the samples and can be utilised to distinguish root bark samples based on chemical marker content. Two chemotypes were identified in the samples. Sericoside was associated with Chemotype 1, while sericic acid was associated with Chemotype 2. Further studies to distinguish the chemotypes based on their activities towards specific targets would assist in chemotype selection for commercial use. The study has demonstrated that HPTLC alone, and in combination with chemometrics, is a powerful tool that can be applied to the QC of *T. sericea* root. The study has also revealed that the use of white light reflectance to view HPTLC plates in metabolomic analysis has limitations, but these can be overcome through careful inspection and interpretation of pixel amplitudes on the corresponding densitograms of individual samples with known concentrations of marker compounds.

## Figures and Tables

**Table 1:** Sample concentration with band intensity (Key: faint (+), intense (++), very intense (+++)). The quantitative values for the samples were determined using ultra-performance liquid chromatography with photodiode array detection. The localities where samples were collected are listed, as well as their assigned voucher specimen numbers.

Locality	Voucher	Compound Concentration					
		Resveratrol-3-*O*-*β*-Rutinoside (Brown R*f* = 0.24)	Sericic Acid(Blue R*f* = 0.83)		Sericoside(Blue R*f* = 0.48)		
		mg/g	BI	mg/g	BI	mg/g	BI
**KNP**	CPA004						
K1		24.7	+++	18.3	+++	11.2	++
K2		19.0	++	12.3	++	6.14	+
K3		26.4	+++	18.9	+++	28.4	+++
K4		26.0	+++	20.2	+++	12.6	+++
K5		16.0	++	11.4	++	6.64	++
**Giyani**	CPA002						
G1		17.9	++	3.18	++	8.36	++
G2		26.6	+++	18.6	+++	26.6	+++
G3		25.9	+++	19.0	+++	27.7	+++
G4		11.1	++	3.35	++	7.33	++
**Bela-Bela**	CPA001						
BP1		28.1	+++	18.0	+++	12.4	++
BP2		28.0	+++	17.0	+++	26.3	+++
BP3		27.9	+++	2.58	+	4.82	+
**Tzaneen**	CPA009						
TZ1		3.03	+	15.9	+++	30.2	+++
TZ2		2.22	+	18.2	+++	25.4	+++
TZ3		16.7	++	19.0	+++	27.2	+++
TZ4		27.9	+++	17.4	+++	26.1	+++
TZ5		2.23	+	4.81	+	15.4	++
**Tshandama**	CP007						
TSA1		27.7	+++	18.7	+++	28.0	+++
TSA2		27.5	+++	22.7	+++	27.0	+++
TSA3		29.0	+++	19.7	+++	26.9	+++
TSA4		28.9	+++	18.9	+++	28.6	+++
TSA5		29.0	+++	19.2	+++	27.6	+++
**Tshitavha**	CPA008						
TSH1		28.8	+++	17.7	+++	28.4	+++
TSH2		29.0	+++	18.0	+++	27.4	+++
TSH3		17.7	++	18.4	+++	28.3	+++
TSH4		27.8	+++	19.5	+++	27.2	+++
TSH5		28.4	+++	19.6	+++	28.7	+++
**Mookgophong**	CPA006						
MP1		26.9	+++	8.36	++	15.4	++
MP2		27.7	+++	16.6	+++	17.2	++
MP3		29.8	+++	18.0	+++	28.7	+++
MP4		15.4	++	5.25	+	28.7	+
MP5		18.3	++	5.02	+	7.55	+
**Vuwani**	CPA010						
V1		16.7	++	7.36	++	6.89	+
V2		18.1	++	8.69	++	15.7	++
**Maila**	CPA003						
J1		1.54	+	3.78	++	27.0	+++
J2		1.43	+	3.55	+++	24.4	+++
J3		2.05	++	18.3	+++	24.5	+++
J4		2.65	+	19.0	+++	26.0	+++
**Mavhambe**	CPA005						
MMR1		25.4	+++	3.77	++	12.8	++
MMR2		27.6	+++	6.23	++	26.6	+++
MMR3		13.4	++	7.48	++	13.7	++
MMR4		16.2	++	7.36	++	13.7	+++

R*f* = retardation factor; KNP = vicinity of Kruger National Park; B1 = band intensity.

## Data Availability

The data presented in this study are available on request from the corresponding author.

## Supplementary Materials

The following are available online. Figure S1: (A) Principal component analysis (PCA) scores plot and (B) Loadings scores plot obtained from the HCA and PCA (UPLC-MS data) analysis of *T. sericea* root samples, Table S1: UPLC-PDA method validation results for the quantitative determination of resveratrol-3-*O*-*β*-rutinoside, sericic acid and sericoside in 42 *T. sericea* root bark samples.

## Sample Availability:

Samples of the compounds resveratrol-3-*O*-*β*-rutinoside, sericic acid and sericoside are available from the corresponding author.
