# Supplementary material for: Phytochemical Profiling and Quality Control of Terminalia sericea Burch. ex DC. Using HPTLC Metabolomics
Source: Molecules. 2021 Jan 15;26(2):432. doi: 10.3390/molecules26020432 (PMC7830210; doi:10.3390/molecules26020432)
Supplement: Supplementary file 1 [file molecules-26-00432-s001.pdf]

SUPPLEMENTARY MATERIAL

Phytochemical profiling and quality control of *Terminalia sericea* Burch. ex DC. using HPTLC metabolomics

Nduvho Mulaudzi[1], Chinedu P Anokwuru[1], Sidonie Y. Tankeu[1], Sandra Combrinck[1], Weiyang Chen[1], Ilze Vermaak[1,2], Alvaro M Viljoen[1,2],*

[1]Department of Pharmaceutical Sciences, Faculty of Science, Tshwane University of Technology, Private Bag X680, Pretoria 0001, South Africa.
[2]SAMRC Herbal Drugs Research Unit, Faculty of Science, Tshwane University of Technology, Private Bag X680, Pretoria 0001, South Africa.
*Corresponding author:
Alvaro Viljoen
Department of Pharmaceutical Sciences
Faculty of Science
Tshwane University of Technology
Private Bag X680, Pretoria, 0001, South Africa.
Email: viljoenam@tut.ac.za
Tel: +27 12 382 6373
Fax: +27 12 382 6243

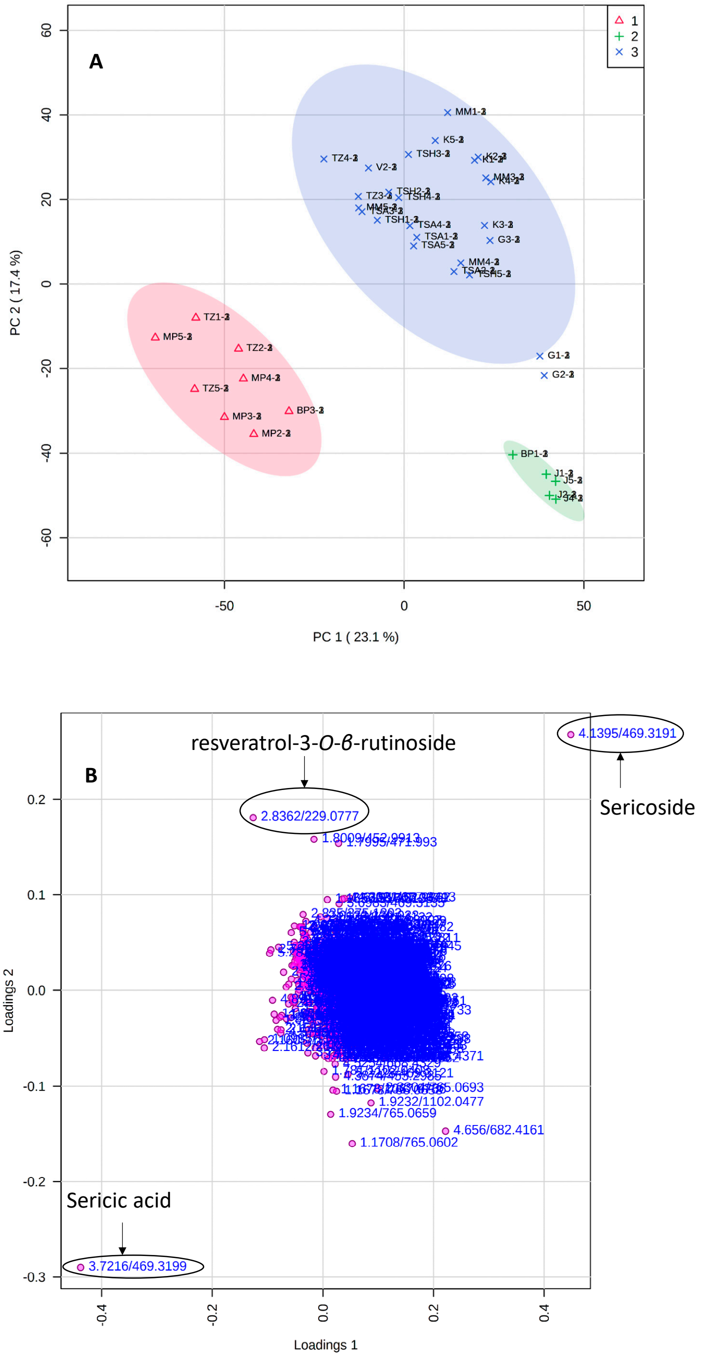

Figure S1: A) Principal component analysis (PCA) scores plot and B) Loadings scores plot obtained from UPLC-MS data (N = 39) of samples collected from Waterberg, Mopani and Vhembe districts of Limpopo Province. Red: samples from Waterberg and Mopani; green: samples from Waterberg and Vhembe; blue: samples from Mopani and Vhembe districts.

Table S1: Regression equation, coefficient of determination (R^2), limits of detection (LOD) and quantification (LOQ), accuracy and intra-and interday precision, as determined for the UPLC-PDA method of analysis for each of the major constituents of *T. sericea* root.

Compound	Regression equation	R^2	LOD (ng/mL)	LOQ (ng/mL)	Accuracy (%)	Intraday Precision %RSD	Interday Precision %RSD
R-3-R	y =19048x-16578	0.998	23.3	70.8	102	0.60	0.60
Sericic acid	y = 733.4x-166.9	0.998	25.2	76.5	98.3	1.65	1.78
Sericoside	y =851.5x+205.6	0.999	11.6	35.2	99.9	1.00	1.34

R-3-R: resveratrol-3-*O*-*β*-rutinosid
